# Novel Electrospun Polycaprolactone/Calcium Alginate Scaffolds for Skin Tissue Engineering

**DOI:** 10.3390/ma16010136

**Published:** 2022-12-23

**Authors:** Maria I. Echeverria Molina, Chi-An Chen, Jeniree Martinez, Perry Tran, Kyriakos Komvopoulos

**Affiliations:** Department of Mechanical Engineering, University of California, Berkeley, CA 94720, USA

**Keywords:** fiber crosslinking, fibroblasts, hydrophilicity, keratinocytes, electrospun poly(ε-caprolactone)/calcium alginate scaffolds, porosity, skin tissue engineering

## Abstract

After decades of research, fully functional skin regeneration is still a challenge. Skin is a multilayered complex organ exhibiting a cascading healing process affected by various mechanisms. Specifically, nutrients, oxygen, and biochemical signals can lead to specific cell behavior, ultimately conducive to the formation of high-quality tissue. This biomolecular exchange can be tuned through scaffold engineering, one of the leading fields in skin substitutes and equivalents. The principal objective of this investigation was the design, fabrication, and evaluation of a new class of three-dimensional fibrous scaffolds consisting of poly(ε-caprolactone) (PCL)/calcium alginate (CA), with the goal to induce keratinocyte differentiation through the action of calcium leaching. Scaffolds fabricated by electrospinning using a PCL/sodium alginate solution were treated by immersion in a calcium chloride solution to replace alginate-linked sodium ions by calcium ions. This treatment not only provided ion replacement, but also induced fiber crosslinking. The scaffold morphology was examined by scanning electron microscopy and systematically assessed by measurements of the pore size and the diameter, alignment, and crosslinking of the fibers. The hydrophilicity of the scaffolds was quantified by contact angle measurements and was correlated to the augmentation of cell attachment in the presence of CA. The in vitro performance of the scaffolds was investigated by seeding and staining fibroblasts and keratinocytes and using differentiation markers to detect the evolution of basal, spinous, and granular keratinocytes. The results of this study illuminate the potential of the PCL/CA scaffolds for tissue engineering and suggest that calcium leaching out from the scaffolds might have contributed to the development of a desirable biological environment for the attachment, proliferation, and differentiation of the main skin cells (i.e., fibroblasts and keratinocytes).

## 1. Introduction

Skin, the largest organ in the human body, is a multilayered and complex structure consisting of three characteristic layers—epidermis, dermis, and hypodermis. While fibroblasts are the main cells in the dermis, keratinocytes primarily make up the three sublayers of the epidermis [1]. The inner layer comprises basal keratinocytes interfaced with the fibroblasts, followed by two layers consisting of spinous and granular keratinocytes [1,2]. This organization also corresponds to the order of differentiation of these cells, with granular derived from spinous and spinous derived from basal keratinocytes. In this cellular arrangement, macronutrients (i.e., glucose, amino acids, and lipids) and micronutrients (e.g., vitamins A, C, D, and E, zinc, calcium, copper, and selenium) act to modulate skin health and function [3]. Specifically, calcium gradients through the epidermis play an important role in regulating sequential differentiation of the keratinocytes. The increase in calcium proliferates the formation of desmosomes, which are adhesion junctions between cells playing an important role in different signaling pathways critical for differentiation [4]. An upsurge in calcium concentration also leads to the migration of lamellar bodies to the apex of the uppermost cells in the granular layer, where lipids secreted by the lamellar bodies are arranged within intercellular spaces, contributing to the hydrophobic matrix responsible for the waterproofing capability of the skin permeability barrier [5].

The skin is the first line of defense against outside pathogens and the environment (e.g., microorganisms, radiation, and chemicals), providing temperature regulation, sensory functions, autonomic functions (exocrine secretion), and endocrine regulation [6]. Being the outermost organ of the body, the skin can be easily injured or damaged. Normal wound healing of skin encompasses a continuous cascading process including inflammation, cell proliferation and migration, and remodeling. While the wound healing process is very effective in the case of superficial injuries, the process becomes impaired for deep and chronic wounds requiring further treatment due to various inhibiting factors such as bacterial invasion, comorbidities, medications, and lifestyle habits [7,8]. Therefore, deep-wound healing is an intricate process whose treatment and management are still challenging despite decades of research and multiple options for skin replacement and regeneration such as allografts, autografts, and skin substitutes [9,10]. These challenges are mainly associated with the integration and rejection of grafts, infection, scar contraction, and growth of not fully functional skin tissue [9,11].

Scaffolds have played a critical role in tissue regeneration, with past research leading to potential success in various tissue applications such as cardiac muscle [12], bone [13], liver [14], and skin [15,16]. Scaffolds are three-dimensional (3D) networks made of different materials by various fabrication processes including decellularized extracellular membranes [17,18], freeze drying [19], electrospinning [20], and 3D printing [16]. Particularly, electrospinning is a widely used fabrication method that uses electrostatic force to generate a charged stream of a polymeric solution from a liquid droplet, which stretches and elongates to generate fibers. Fibrous membranes of tunable fiber diameter, porosity, pore size, and fiber alignment can be easily fabricated due to the simplicity and flexibility of the electrospinning process [21]. Most of the materials used are biodegradable polymers encompassing synthetic, natural, or combinations of the former polymers (i.e., composites and co-polymers) [22,23]. Some of the most common materials used to fabricate scaffolds for tissue engineering are poly(L-lactic acid) (PLLA), poly(glycolic acid) (PGA), and poly(ε-caprolactone) (PCL). The physical and mechanical properties of scaffolds can be tuned to elicit specific cellular responses by modifying their microstructure (i.e., fiber diameter, pore size, and porosity) and chemistry (e.g., co-polymers and motifs) [23]. Since cellular interactions are critical for wound healing, specifically keratinocytes and fibroblasts, a potential successful treatment for skin healing must not only provide structural and biological support to the cells, but also allow for the exchange and arrangement of nutrients, oxygen, and biochemical signals in order to stimulate cell proliferation and differentiation.

PCL is a well-known biocompatible material consisting of semicrystalline polyester, which can be readily degraded by lipases and esterases. The regular structure of PCL comprises linear carbon–carbon bonds, which are responsible for its relatively low melting point (~60 °C) [24] and low glass transition temperature (about −60 °C) [25], resulting in a stable semicrystalline structure that exhibits high strength in the human body due to the amorphous domains existing in the rubbery state. However, because the PCL structure contains hydrophobic –CH_2_ moieties and does not have any side chains, it degrades hydrolytically at a slower rate than other synthetic polymers (e.g., the order of degradation rate is PCL < PLLA < PGA) [26,27]. Nevertheless, PCL is a desirable biomaterial for scaffold engineering because its properties can be adjusted according to the application requirements by blending with other materials [28]. Alternatively, calcium alginate (CA) is a hydrogel possessing high hydrophilicity, high water absorption capacity, and good biocompatibility. Because alginate-based materials exhibit a hemostatic effect, they are often used as wound healing agents in wound dressings [29]. CA is brittle and its mechanical properties depend on the divalent cation (Ca^2+^) covalent linkages to the alginate to form a 3D structure, referred to as “egg-box” [29,30,31]. Alginate has the unique property of integrating well with other polymers. Specifically, incorporating CA in PCL enables the modification of the hydrophilicity, degradation rate, and mechanical strength, enabling specific cellular behaviors to be elicited, as reported in previous research concerned with emulsions of synthetic polymers and alginates [32,33,34,35].

In this study, a PCL/sodium alginate (SA) solution was used to fabricate fibrous scaffolds by electrospinning. The produced scaffolds were then treated with a calcium chloride solution to replace the sodium ions from the alginate with calcium ions, resulting in the formation of PCL/CA scaffolds. The principal objective was to enhance skin tissue regeneration through the release of calcium ions from the PCL/CA scaffolds into the biological media to stimulate keratinocyte differentiation. Subsequently, the microstructure, hydrophilicity, and in vitro behavior of scaffolds seeded with keratinocytes and fibroblasts were examined in light of the experimental results. Keratinocyte differentiation was investigated by immunofluorescence staining. Furthermore, adhesion and proliferation of the keratinocyte and fibroblast cells were evaluated to assess the pro-regenerative capacity of the scaffolds for a fully functional epidermis layer. The performance of these scaffolds was compared to similar constructs fabricated by electrospinning using a PCL/SPAN 80 solution (control scaffolds). The results presented below reveal that calcium leaching out from the PCL/CA scaffolds augments the behavior of the cells seeded on these scaffolds.

## 2. Materials and Methods

### 2.1. Experimental Materials

The electrospinning solution was an emulsion, with the continuous phase being an oil phase consisting of PCL (80,000 Da) dissolved in a dichloromethane (DCM) and sorbitan monooleate (SPAN 80) surfactant (all from Sigma-Aldrich, St. Louis, MO, USA). The PCL was dissolved in DCM at a concentration of 8% *w*/*w* and sonicated for 30 min before adding SPAN 80 at 17% of the PCL weight. The water phase was a discontinuous phase of SA (Sigma-Aldrich, St. Louis, MO, USA) prepared by dissolving SA in DI water at a concentration of 40 mg/mL for 24 h at room temperature. The water phase was combined with the oil phase and vortexed for 10 min, then sonicated for 30 min, and finally vortexed for 10 min at room temperature. The emulsion was electrospun immediately after preparation.

### 2.2. Scaffold Fabrication

Fibrous scaffolds with a thickness ~200 µm were fabricated with an electrospinning setup equipped with a parallel disk collector, described in detail elsewhere [20,36,37]. The PCL solution was pumped at a rate of 0.8 mL/h through a 22 G flat-tip needle affixed 10 cm above the collector axis. In all experiments, the potential difference between the needle and the collector was set at 20 kV. To ensure uniform fiber deposition during electrospinning, the collector was rotated at 8.4 rpm. The collector design entails two parallel aluminum plates attached to a 1.5-cm-diameter shaft and placed at a lateral distance of 1.5 cm from each other. This collector geometry yields bilayer scaffolds with morphologies characterized by fibers mostly aligned between the parallel plates (bottom surface) and randomly oriented fibers (top surface) [36,37].

### 2.3. Scaffold Post-Treatment

The SA was transformed to CA by immersing the scaffolds in an ethanol solution (Decon Labs, King of Prussia, PA, USA) with 2 *w*/*w*% calcium chloride (Sigma-Aldrich, St. Louis, MO, USA) for 2 h. The purpose of this treatment was to modify the morphology (i.e., fiber crosslinking) and the chemical characteristics of the scaffolds, and also to study how the cell activity was influenced by these modifications. Hereafter, the PCL/SPAN 80 scaffolds will be referred to as the control scaffolds, whereas the post-treated scaffolds will be referred to as the PCL/CA scaffolds, for brevity.

### 2.4. Characterization Techniques

The surface and cross-sectional morphologies of the control and PCL/CA scaffolds were examined with a field-emission scanning electron microscope (SEM) (TM-4000, Hitachi, Tokyo, Japan) operated at an acceleration voltage of 15 kV. SEM images were used to determine the fiber diameter and the pore size distribution in the scaffolds. The fiber diameter was determined by randomly selecting 30 fibers from the top and the bottom surfaces of three similar scaffolds using ImageJ software (version 1.53e, National Institute of Health, Bethesda, MD, USA). The scaffold pore size was estimated with ImageJ software using the analyze particle function. The scaffold thickness was measured from cross sections obtained by cutting the scaffolds with ultrasharp platinum-coated blades. The analysis of the fiber diameter and the porosity was performed with 500× magnification SEM images, whereas the scaffold thickness was measured from 200× magnification SEM images.

The hydrophilicity of the control and PCL/CA scaffolds was quantified by dynamic contact angle measurements obtained by depositing a 5 μL DI water droplet at the top surface of the scaffolds. DSA software (KRUSS, Hamburg, Germany) was then used to record a 2-min video of 3600 frames. The start time was set as the moment the droplet separated from the pipette tip to deposit onto the scaffold surface. Then, the contact angle of each sample was measured every 5 s for a total of 35 s using the low bond axisymmetric drop shape analysis (LBADSA) plugin (Biomedical Imaging Group, Lausanne, Switzerland) in ImageJ software. A total of 3–5 samples were used in the contact angle measurements.

The fiber orientation at the top and bottom surfaces of the scaffolds was analyzed with a modified fast Fourier transform (FFT) technique detailed elsewhere [36,38]. SEM images (2500× magnification) were converted to grayscale 8-bit images. ImageJ software (version 1.53e, National Institute of Health, Bethesda, MD, USA) with oval profile plugin [39] was used to complete the 2D FFT analysis. The data obtained with this method were normalized to a zero baseline.

### 2.5. In Vitro Experiments

**Figure 1:**
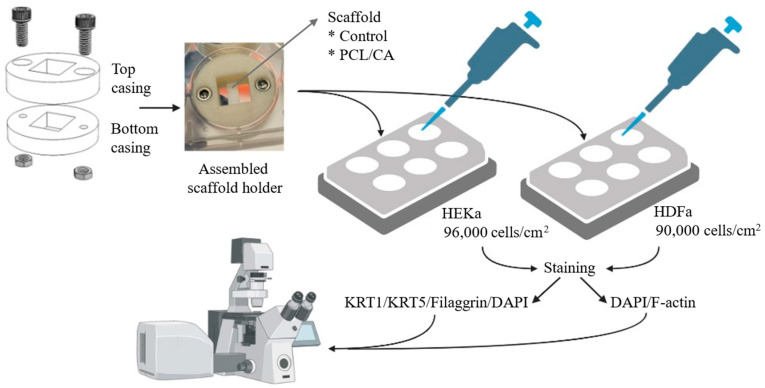
The roadmap to in vitro testing of the present study (the figure was created with BioRender).

For optimal cell growth, the scaffolds were suspended in the media by special sample holders (Figure 1), inspired by a previous study [40]. The sample holder assembly, fabricated by 3D printing (F170, Stratasys, Eden Prairie, MN, USA) using acrylonitrile butadiene styrene (ABS-M30™, Stratasys, Eden Prairie, MN, USA), consisted of two parts with a central rectangular cut-out screwed together by recessed stainless-steel screws and nuts. The diameter of the holder was 33 mm (i.e., slightly smaller than the diameter of the six-well plates used in the in vitro experiments). The height of the top and the bottom casing was 8 and 6 mm, respectively, whereas the central cut-out area was 14 mm × 12 mm.

Before the cell culture, the sample holders and all other assembly components (i.e., tweezers, Allen wrench, etc.) were sterilized by immersion in 95% ethanol (Decon Labs, King of Prussia, PA, USA) for 24 h. Scaffolds cut to a 5 mm width and 15 mm length and sterilized with ethanol were placed on glass slides and air-dried inside a biosafety hood (NV25-600, NuAire, Plymouth, MN, USA) equipped with UV light. To maintain a sterile environment, both the assembly of the sample holders and clamping of the scaffolds between the casings were performed in a large Petri dish (152 cm^2^) inside a biosafety hood. The bottom surface of the scaffold was placed facing the bottom of the well. The scaffold area exposed to the cells was 14 mm × 5 mm.

To assess the potential of the scaffolds for skin tissue engineering, in vitro testing was performed with primary normal human dermal fibroblast from adults (HDFa) cells (PCS-201-012, ATCC, Manassas, VA, USA) and primary normal human epidermal keratinocytes from adults (HEKa) cells (PCS-200-011, ATCC, Manassas, VA, USA). The cells were cultured using a mixture of keratinocyte growth kit (PCS-200-040, ATCC, Manassas, VA, USA) and basal medium (PCS-200-030, ATCC, Manassas, VA, USA) for the keratinocytes and a mixture of fibroblast growth kit (PCS-201-040, ATCC, Manassas, VA, USA) and basal medium (PCS-201-030, ATCC, Manassas, VA, USA) for the fibroblasts. Both cell lines were grown and maintained in their respective media in a humidified incubator of 37 °C and 5% CO_2_ atmosphere. During scaffold cell seeding, each medium was supplemented with 1% streptomycin (Thermo Fisher Scientific, Pittsburgh, PA, USA).

Control and PCL/CA scaffolds were seeded with HDFa and HEKa cells on two plates each having six wells. Specifically, four samples were seeded with HEKa cells (density = 96,000 cells/cm^2^) on one of the plates and four samples were seeded with HDFa cells (density = 90,000 cells/cm^2^) on the other plate. In both cases, the cells were allowed 4 days to attach to the scaffolds and 9 more days to proliferate. Table 1 summarizes the well-plate arrangements of the test scaffolds, and Figure 1 shows the roadmap to the in vitro experiments. After a total of 13 days since the instigation of cell seeding, the scaffolds were fixed with 4% paraformaldehyde (Electron Microscopy Sciences, Hatfield, PA, USA) and treated with 0.1% Triton X-100 (Fisher Scientific, Fair Lawn, NJ, USA) and 1% bovine serum albumin (Sigma-Aldrich, St. Louis, MO, USA). The antibody staining was a two-day process. On the first day, the fixed scaffolds with HEKa cells were stained with KRT5 mouse monoclonal antibody (Sigma-Aldrich, St. Louis, MO, USA), cytokeratin 1 (KRT1) guinea pig polyclonal antibody (OriGene Technologies, Rockville, MD, USA), and filaggrin rabbit polyclonal antibody (Invitrogen™, Thermo Fisher Scientific, Pittsburgh, PA, USA) and stored at 4 °C overnight. On the second day, rabbit anti-mouse IgG (H + L) highly cross-adsorbed secondary antibody, Alexa Fluor™ Plus 555 (Invitrogen™, Thermo Fisher Scientific, Pittsburgh, PA, USA), goat anti-guinea pig IgG (H + L) highly cross-adsorbed secondary antibody, Alexa Fluor™ 488 (Thermo Fisher Scientific, Pittsburgh, PA, USA), goat anti-rabbit IgG (H + L) cross-adsorbed secondary antibody, Alexa Fluor™ 633 (Thermo Fisher Scientific, Pittsburgh, PA, USA), and DAPI (MilliporeSigma, Burlington, MA, USA) were added to the fixed scaffolds. Scaffolds seeded with the HDFa cells were only stained with DAPI and F-actin (Alexa Fluor™ 488, Invitrogen™, Thermo Fisher Scientific, Pittsburgh, PA, USA) on the second day. Cell attachment, proliferation, infiltration depth, and differentiation were studied with a laser scanning confocal microscope (Carl Zeiss Microscopy, Oberkochen, Germany). Table 2 summarizes the antibodies and the dilution/concentration used in the present study.

### 2.6. Statistical Methods

The acquired data of the fiber diameter, pore size, and infiltration depth were analyzed with one-way ANOVA using Stata/SE 16.1. The contact angle data were plotted as mean values and error bars corresponding to one standard deviation above and below the corresponding mean value. The box plots and histograms presented in the next section were created with Stata/SE 16.1.

## 3. Results

### 3.1. Scaffold Morphology and Hydrophilicity

**Figure 2:**
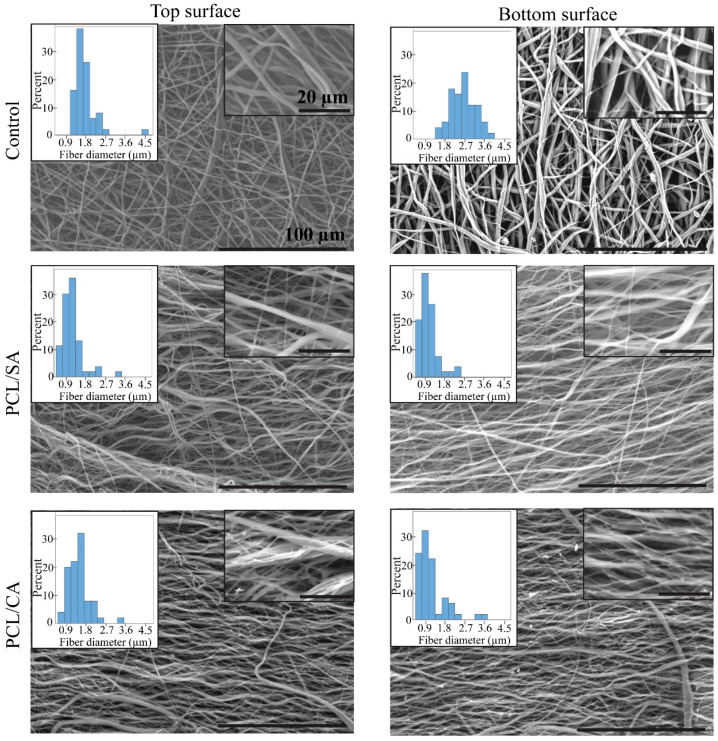
SEM images showing the morphology of the top and bottom surfaces of control, PCL/SA, and PCL/CA scaffolds. The scale bars in all insets on the right and main images are equal to 20 and 100 µm, respectively. The insets on the left show the fiber diameter histogram of each scaffold.

**Figure 3:**
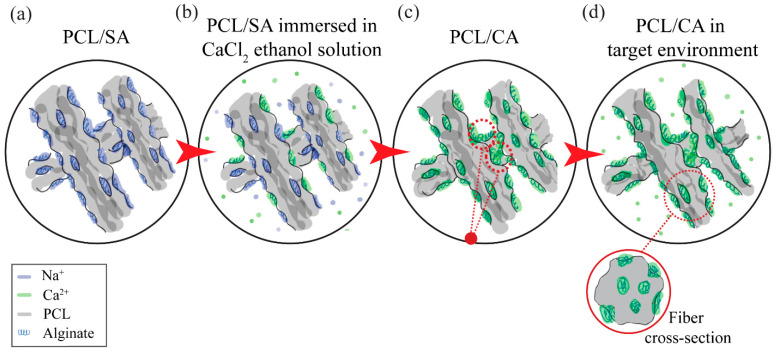
Schematic illustration of the leaching out process of the Ca^2+^ ions. (**a**) As-fabricated PCL/SA scaffold, (**b**) scaffold immersion in a CaCl_2_ ethanol solution triggers three different processes—calcium diffusion into the scaffold, sodium leaching out from the fibers, and calcium alginate acting as a crosslinker resulting in (**c**) a scaffold consisting of only PCL/CA fibers, which in the target environment (**d**) demonstrates Ca^2+^ ion leaching out from its surface and the bulk of the fibers.

Figure 2 shows representative SEM images of the surface morphology of the control, PCL/SA, and PCL/CA scaffolds. All images show the formation of continuous fibers having a fairly uniform diameter. The PCL/CA scaffolds display evidence of notable fiber crosslinking, indicated by the fiber entanglement and fusion at multiple points, as depicted in the SEM images shown in the right insets of Figure 2. Fiber crosslinking occurred during the immersion in the ethanol solution that contained calcium chloride through the replacement of the Na^+^ ions in the SA by the Ca^2+^ ions of the solution. This ion exchange is a well-documented biomolecular phenomenon, often referred to as the egg-box model [30,31]. During the immersion in the target environment (i.e., the cell culture media), the Ca^2+^ ions in the PCL/CA scaffolds leached out both from the surface and the interior of the fibers by a process schematically depicted in Figure 3, making the Ca^2+^ ions readily available for the cells to metabolize. Similar observations (i.e., fiber uniformity and crosslinking) were made for the through-thickness scaffold morphology.

**Figure 4:**
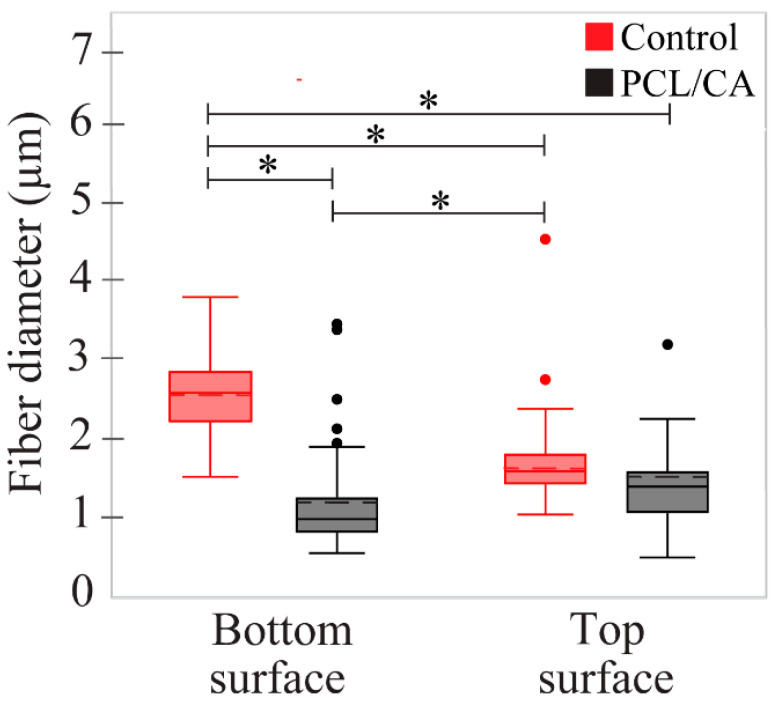
Statistical results of the fiber diameter at the top and bottom surfaces of the control and PCL/CA scaffolds. The dashed lines inside the boxes represent the means of each dataset. (*) Indicates a statistically significant difference with a confidence level of 95% (*p* < 0.05).

The bottom and top surfaces of the control scaffolds displayed a mean fiber diameter of 2.57 and 1.72 μm, respectively, whereas the bottom and top surfaces of the crosslinked PCL/CA scaffolds exhibited thinner fibers with a mean diameter equal to 1.21 and 1.59 μm, respectively. The left insets of Figure 2 show the fiber diameter distributions. Statistical results of the fiber diameter at the bottom and top surfaces of the control and PCL/CA scaffolds are presented in Figure 4.

**Figure 5:**
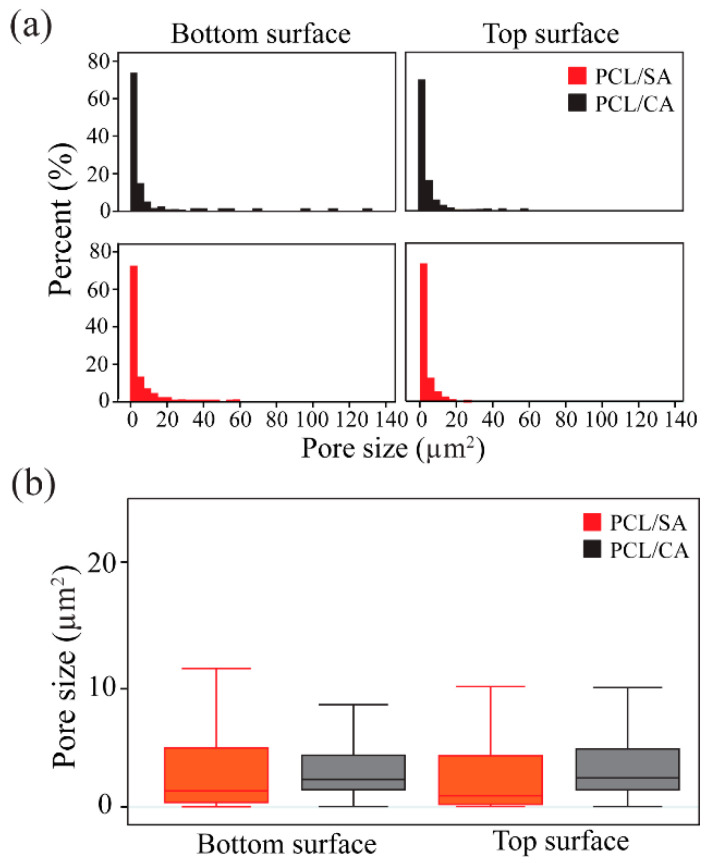
(**a**) Distributions and (**b**) statistical results of the estimated pore size at the top and bottom surfaces of the PCL/SA (uncrosslinked) and PCL/CA (crosslinked) scaffolds. The data suggest that fiber crosslinking resulted in the formation of more and smaller pores. Differences between the means of the datasets are not statistically significant (*p* > 0.05). The outliers of the box plots shown in (**b**) are not shown for clarity.

Figure 5 shows the results of the estimated pore size at the bottom and top surfaces of the uncrosslinked PCL/SA and crosslinked PCL/CA scaffolds. The pore size distribution plots (Figure 5a) revealed slightly smaller pores at the bottom surfaces than the top surfaces of the PCL/SA and PCL/CA scaffolds. The fact that crosslinking contributed to the formation of additional pores is evidenced by the slightly higher percentage of small pores in the distribution plots (Figure 5a) and the slightly narrower dataset, particularly at the bottom surface of the PCL/CA scaffold (Figure 5b). However, there was no statistically significant difference between the pore size means (*p* > 0.05) of the bottom and top surfaces of the PCL/SA and PCL/CA scaffolds (Figure 5b). The apparent decrease in pore size may be attributed to the fact that the relatively thinner fibers at the bottom surface of the PCL/CA scaffolds increased both the surface area and the number of crosslink sites, also contributing to the fiber fusion and entanglement.

**Figure 6:**
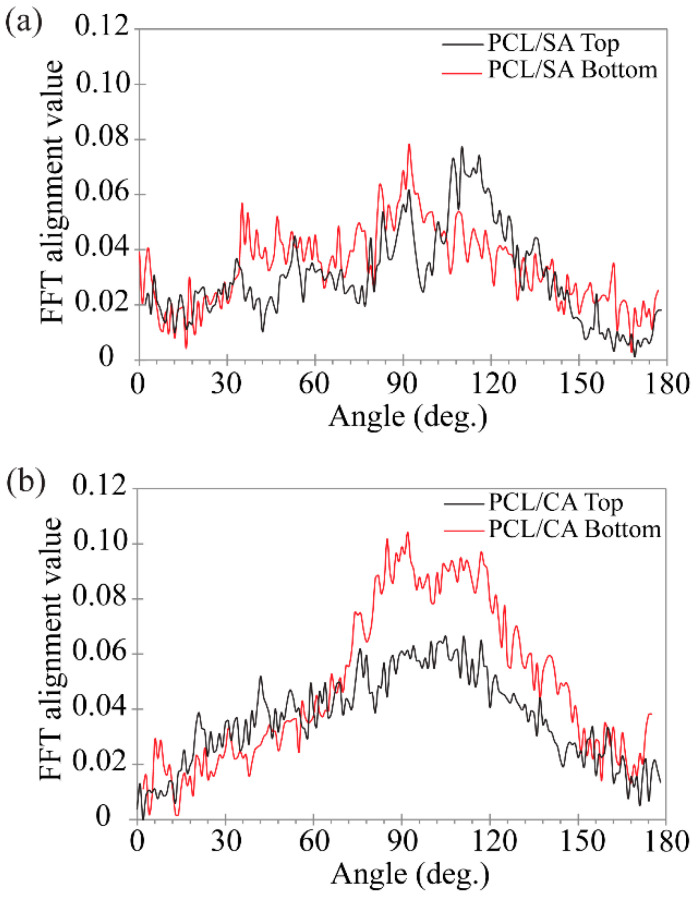
Representative distributions of the fiber alignment at the top and bottom surfaces of the (**a**) PCL/SA and (**b**) PCL/CA scaffolds.

The fiber morphology of the PCL/SA and PCL/CA scaffolds was further studied with a modified FFT technique. While the PCL/SA scaffold surfaces did not demonstrate a dominant direction of fiber alignment (Figure 6a), the PCL/CA scaffolds revealed that the crosslinking process yielded a more ordered morphology characterized by planar fiber alignment, especially at the bottom surface (Figure 6b). The wide distributions of fiber alignment indicate that there is not a specific angle of alignment in the PCL/CA scaffolds, but a wide range of alignment directions.

**Figure 7:**
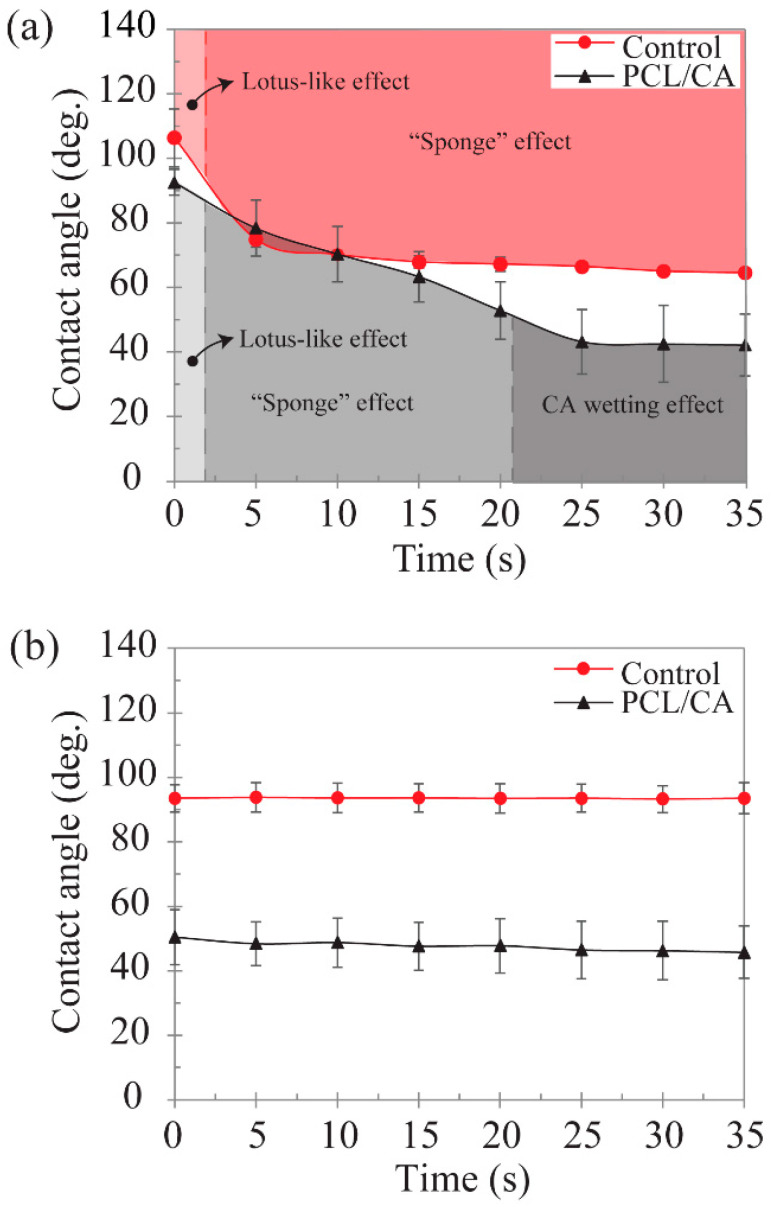
Time-dependent contact angle measured at the top surface of the control and PCL/CA samples: (**a**) electrospun fibrous scaffolds and (**b**) spin-coated membranes. Data points represent mean values of 3–5 measurements. Error bars represent one standard deviation above and below the corresponding mean value.

Dynamic contact angle measurements provided insight into the hydrophilicity of the scaffolds and their potency to elicit cell attachment. All measurements were performed at the top surface of the scaffolds. The control scaffolds displayed an initial contact angle of 106°, which is indicative of a hydrophobic behavior, whereas the PCL/CA scaffolds showed an initial contact angle of 92°, which classifies these scaffold surfaces as borderline hydrophilic (Figure 7a). However, both types of scaffolds exhibited a rapid decrease in contact angle in the first 20 s, with a slower decrease commencing afterward. Specifically, the contact angle of the control scaffolds decreased below 90° and eventually stabilized at ~70°, suggesting hydrophilic steady-state characteristics, whereas the contact angle of the PCL/CA scaffolds exhibited an even more dramatic decrease to a steady-state value of ~45°. To further investigate this trend and exclude the possible effects of the scaffold morphology, contact angle measurements were obtained from spin-coated solid membranes of the control and PCL/CA solutions (Figure 7b). These assays demonstrated a fairly steady contact angle of ~90° and ~45° for the control and PCL/CA samples, respectively, both suggesting a hydrophilic behavior. Thus, the PCL/CA scaffolds were clearly more hydrophilic than the control scaffolds, even when comparing a spin-coated alginate solution to an electrospun control scaffold. Since the surfaces of the spin-coated membranes were much smoother than those of the electrospun fibrous scaffolds, it may be inferred that the contact angle of the membrane samples was mostly affected by surface chemistry. The results shown in Figure 7 indicate that the addition of CA greatly augmented the hydrophilicity of the polymer solution, suggesting an increased latency for cell attachment.

The time-dependent variation in the dynamic contact angle of the control and PCL/CA scaffolds (Figure 7a) may be associated with the dominance of different mechanisms. Initially, both groups demonstrated a lotus-like effect [41], with large contact angles measured at the inception of testing. The rapid decrease in the contact angle of the control scaffolds to a stable angle of ~70° was attributed to the entrapment of air in the larger pores of this scaffold, which slowed down the wetting process until the water fully occupied the pore area (“sponge” effect). While this phenomenon was also encountered with the PCL/CA scaffolds, the decrease in the contact angle was more pronounced and a stable value was obtained after a longer time compared to the control scaffolds. This was attributed to the latent time of the CA in the PCL/CA scaffolds to react with the water and acquire a gel-like state, driving water transport through the scaffold via water–water and water–CA surface interactions, which forced the air out of the scaffold until the pores were totally occupied by water, consequently resulting in a stable contact angle (CA wetting effect). While the wetting characteristics of the control group were initially controlled by the lotus-like effect and afterward by the “sponge” effect, those of the electrospun PCL/CA scaffolds were sequentially affected by the lotus-like effect, the “sponge” effect, and the CA wetting effect (Figure 7a). The significantly lower steady-state contact angle of the PCL/CA scaffolds than that of the control scaffolds denotes a profound enhancement of the hydrophilicity due to the CA leaching out from the PCL/CA scaffold.

### 3.2. In Vitro Scaffold Characteristics

**Figure 8:**
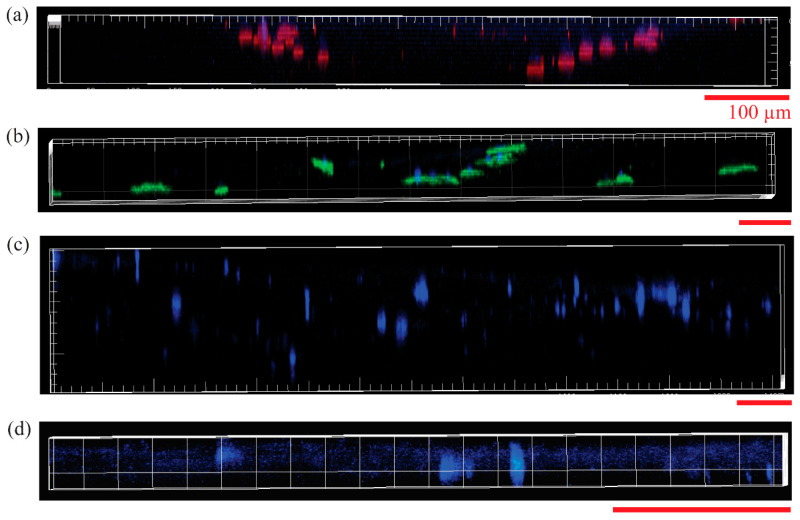
Confocal microscope images showing the infiltration of HEKa cells seeded onto (**a**) control and (**b**) PCL/CA scaffolds, and HDFa cells seeded onto (**c**) control and (**d**) PCL/CA scaffolds (KRT1 (green), KRT5 (red), filaggrin (purple), and DAPI (blue)). All scale bars correspond to 100 µm and are applicable for the horizontal and vertical axes of the images. The antibodies shown in (**a**,**b**) do not indicate exclusivity of expression; these images are only used to visualize the infiltration depth.

**Figure 9:**
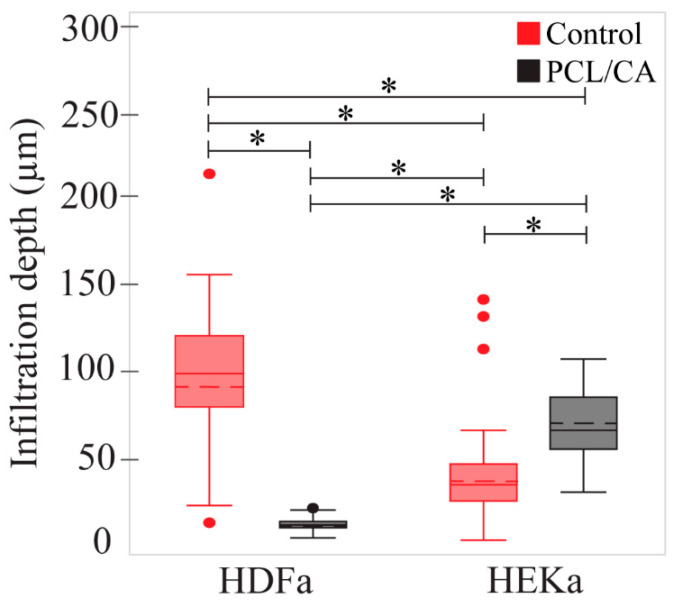
Statistical results of the infiltration depth of the HDFa and HEKa cells through the control and PCL/CA scaffolds. The dashed lines inside the boxes represent the means of each dataset. (*) Indicates a statistically significant difference with a confidence level of 95% (*p* < 0.05).

HDFa and HEKa cells were seeded on control scaffolds in their designated media in two plates and two separate plates on the PCL/CA scaffolds. The cells were allowed 4 days to attach to the scaffolds before changing their media. All cells that did not attach to the scaffolds were aspirated when their media were changed. At 4 days, the seeding cell density was maximized for the volume of media in each well (3 mL). If the media were left for too long, they became acidic due to the CO_2_ released from the cells, affecting the vitality of the cells. The total time of the scaffolds in the incubator was ~9 days. Cell seeding under the aforementioned conditions led to HEKa cell infiltration to a depth of 41.05 ± 29.28 μm (Figure 8a) for the control scaffolds and 68.81 ± 21.99 μm for the PCL/CA scaffolds (Figure 8b). Alternatively, the HDFa cells infiltrated in the control scaffolds to a depth of 92.62 ± 48.57 μm (Figure 8c) and only 12.97 ± 4.48 μm in the PCL/CA scaffolds (Figure 8d). Statistical results of the infiltration depths of HEKa and HDFa cells in the control and PCL/CA scaffolds are presented in Figure 9. The underlying reason for this difference may be the different cell affinity of the keratinocyte and fibroblast cells for the chemistry of the PCL/CA scaffolds. Further research is needed to fully explain the reduced range of fibroblast cell infiltration in the PCL/CA scaffolds.

**Figure 10:**
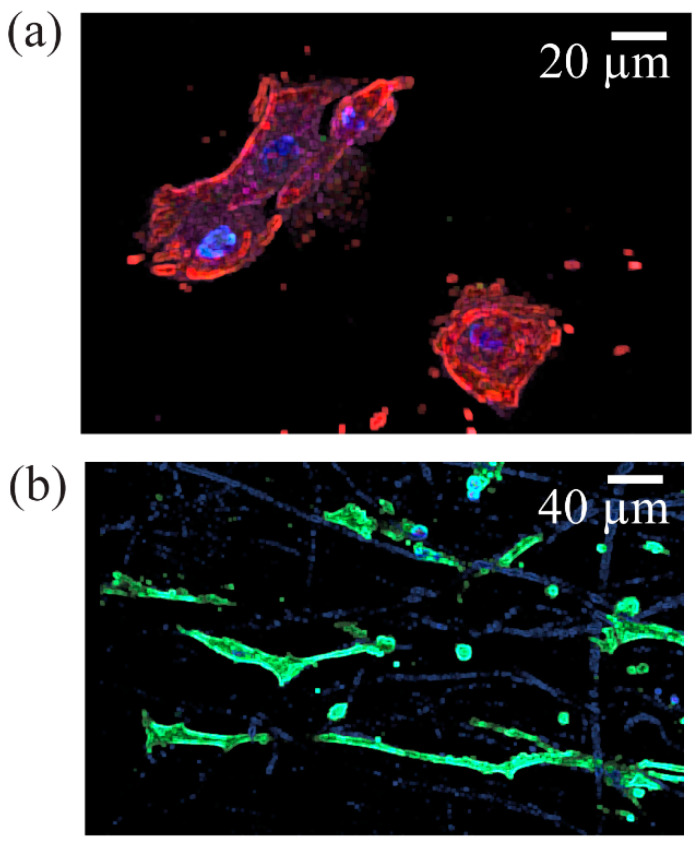
Typical morphology of (**a**) HEKa cells showing a large and round shape (stained image merge of KRT1 (green), KRT5 (red), filaggrin (purple), and DAPI (blue)) and (**b**) HDFa cells showing an elongated shape (DAPI (blue) and F-actin (green)). It appears that HDFa cell attachment occurred preferentially along the fibers of the PCL/CA scaffolds, with some fibers appearing to be stained.

**Figure 11:**
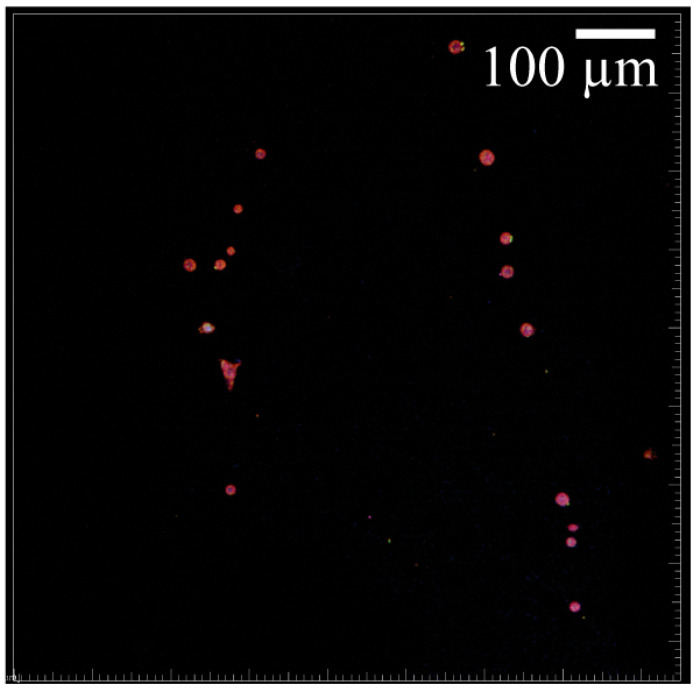
HEKa cells seeded on PCL scaffolds (stained image merge of KRT1 (green), KRT5 (red), filaggrin (purple), and DAPI (blue)) showing cell attachment and alignment along the scaffold fibers.

Cell morphology visualization was aided by differentiation markers KRT5 (red), KRT1 (green), and filaggrin (purple). The HEKa cells seeded on the PCL/CA scaffolds exhibited large and circular configurations (Figure 10a), whereas the HDFa cells seeded on similar scaffolds displayed a typical elongated shape (Figure 10b). Some of the HEKa cells attached and aligned along the fibers of the PCL/CA scaffolds while maintaining a round shape (Figure 11); however, the density of these HEKa cells was not representative of the overall scaffold cell density. HEKa cell differentiation was evident in the confocal images (Figure 8a,b), although no apparent sequential layered differentiation could be observed.

## 4. Discussion

The formation of more and relatively smaller pores at the bottom surface of the PCL/CA scaffolds than the top surface (Figure 5) was attributed to more prominent fiber entanglement due to more pronounced fiber crosslinking (Figure 2). Although the bottom scaffold surface was slightly more porous even before crosslinking, the difference increased after post-treatment with the ethanol solution that contained calcium chloride. The decrease in the fiber diameter at the bottom surface of the crosslinked PCL/CA scaffolds (Figure 4) also contributed to the formation of more and smaller pores. This was ascribed to the increased flexibility of the thinner fibers that facilitated crosslinking, a process depending on close physical proximity and large surface area to generate new connection sites. The increased crosslinking and reduced pore size at the bottom surface might have produced an undesirable effect to cell infiltration through the bottom surface of the PCL/CA scaffolds. Nevertheless, the overall decrease in pore size is consistent with an overall uniform calcium deposition. This is because the decrease in the pore size at the top and bottom surfaces of the PCL/CA scaffolds was a consequence of fiber crosslinking and entanglement (Figure 2) induced by the deposition of Ca^2+^ ions produced from the CA (Figure 3).

The contact angle measurements showed that the PCL/CA scaffolds were more hydrophilic than the control scaffolds, regardless of the fabrication method (i.e., electrospinning and spin-coating) (Figure 7). This can be explained by considering that the control solution only contained an oil phase of DCM, whereas the process requires a water phase to dissolve the alginate. Nonetheless, both solutions yielded sharp drops in contact angle when in scaffold form (Figure 7a), and even the control solution was slightly hydrophilic when spin-coated (Figure 7b). As reported elsewhere [42,43], the contact angle of electrospun PCL is ~100°, which is similar to the initial values of the dynamic contact angle measurements obtained with the control scaffolds, and the ~90° contact angle of spin-coated PCL films measured by others [44]. Although the contact angle tests with the spin-coated membranes suggest the scaffold morphology was responsible for the sudden drop in hydrophilicity, that alone cannot account for the seemingly greater overall hydrophilicity. Among the various factors that might explain this behavior, it is likely that the presence of SPAN 80 in all solutions played a key role. Because SPAN 80 is a surfactant evenly distributed in the polymeric solutions, it may have altered the surface tension [45] between the PCL and the water droplets, consequently reducing the contact angle. It is also possible that the heterogeneous surface morphology of the scaffolds further exacerbated these effects, aiding the adsorption of water onto the scaffold surfaces. It is known that the hydrophobic behavior of PCL limits cell adhesion, migration, proliferation, and differentiation [46]. However, the incorporation of CA in the PCL scaffolds enhanced the hydrophilicity of the material, consequently eliciting a cell behavior conducive of tissue formation, as demonstrated by the in vitro tests (Figure 8, Figure 9, Figure 10 and Figure 11).

The primary purpose for incorporating calcium in the scaffolds was to stimulate keratinocyte differentiation. This is because calcium acts as a major regulator in the epidermis, where the calcium gradient stimulates the differentiation of keratinocytes forming the three epidermal layers (i.e., basal, spinous, and granular layers) [4]. Moreover, understanding the behavior of fibroblasts and keratinocytes seeded on the PCL/CA scaffolds is of utmost importance because dermal-epidermal cross-talk plays an important role in wound healing [47]. The in vitro tests showed that cell attachment and infiltration through the PCL/CA scaffolds were as good or better than the control scaffolds. It was also observed that the seeding time and density affected the attachment and proliferation of both fibroblasts and keratinocytes. Various keratinocyte morphologies were observed (Figure 10a), with the round spinous cells and clusters of cells exhibiting striations, indicating the occurrence of cell differentiation in the electrospun PCL/CA scaffolds. However, a clear pattern of sequential keratinocyte differentiation was not apparent in the cross sections of the PCL/CA scaffolds, in contrast to the findings of a previous study [15], although the constructs used in the foregoing study were fabricated by 3D printing. Alternatively, the control scaffolds demonstrated less cell attachment than the PCL/CA counterparts, reduced cell proliferation, and less obvious keratinocyte differentiation.

As mentioned earlier, some of the keratinocytes seeded on the control scaffolds were found to grow along the fibers (Figure 11). It is possible that the thinner fibers and the decrease in the pore size due to fiber crosslinking in the PCL/CA scaffolds influenced the cell behavior, causing more cell spread. Previous research has confirmed that the scaffold morphology can play an important role in cell behavior, regardless of the scaffold chemistry [23]. Apart from cell attachment and proliferation, the keratinocytes infiltrated deeper into the PCL/CA scaffolds (Figure 8b) compared to the control scaffolds (Figure 8a), also showing increased spread and proliferation. However, an opposite trend was demonstrated by the fibroblasts, with an infiltration depth seemingly larger for the control scaffolds, which requires further examination.

The results of this investigation indicate that the developed PCL/CA scaffolds are promising candidates for skin tissue engineering. The smaller pores at the bottom surface might be advantageous for open wounds where small pores may prevent bacteria from permeating through the scaffolds [48,49] and the fibroblasts to infiltrate and take over spatially-defined structure reserved for the keratinocytes [50,51]. Culture studies with sandwiched PCL/CA scaffolds consisting of two layers separately seeded with keratinocytes and fibroblasts should be of interest to confirm the cross-talk between the two types of cells in the making of new tissue, specifically the epidermal layer and the collagen matrix. The larger pores at the top scaffold surface were found to enhance the ingrowth of both cell types. In addition, the present study provides an impetus for investigating the mechanical properties and in vivo degradation over time of the PCL/CA scaffolds, which is critical to predicting whether these scaffolds possess sufficient strength for the cells to thrive, while creating new extracellular matrix and ultimately new tissue. Although keratinocyte differentiation was observed in the present study, further investigation is needed to confirm that a layered matrix with the form of the native epidermal layer can be produced by a PCL/CA scaffold comprising layers seeded with keratinocytes and fibroblasts.

## 5. Conclusions

Scaffolds consisting of PCL/SPAN 80 and PCL/CA with a continuous fibrous structure were fabricated by electrospinning. The incorporation of CA into the scaffold structure greatly improved the hydrophilicity, promoted fiber crosslinking, reduced the pore size, and enhanced the scaffold capability to elicit the attachment of keratinocyte and fibroblast cells. In addition, CA promoted keratinocyte differentiation, as evidenced from the evolution of different cell morphologies. The non-cytotoxic character of the PCL/CA scaffolds was demonstrated by the proliferation, migration, and infiltration of the cells. The findings of this study illuminate the potential of the present fabrication method to produce skin substitutes from scaffolds that provide a microenvironment and architecture similar to that of the native tissue. Further optimization of key fabrication parameters would unleash the development of scaffolds providing natural micronutrients such as the calcium gradient existing in the human epidermis, boosting the skin regeneration capabilities and the development of transplantable skin substitutes.

## Figures and Tables

**Table 1:** Well-plate arrangements of the test scaffolds.

Cell Type	Media Type	Plate Number	Cell Density (cells/cm^2^)
HDFa	fibroblast media + streptomycin	1	90,000
HEKa	keratinocyte media + streptomycin	2	96,000

**Table 2:** List of antibodies and dilution concentrations used in the cell viability studies.

Primary Antibody	Primary Antibody Dilution	Secondary Antibody	Secondary Antibody Dilution (μL/mL)	Secondary Antibody Stock Concentration (mg/mL)	Keratinocyte Layer
KRT5	1:1000	Alexa Fluor 555	4	2	basal
KRT1	1:200	Alexa Fluor 488	4	2	spinous
Filaggrin	1:1000	Alexa Fluor 633	4	2	granular

## Data Availability

Not applicable.
